# Illuminating Choices for Library Prep: A Comparison of Library Preparation Methods for Whole Genome Sequencing of *Cryptococcus neoformans* Using Illumina HiSeq

**DOI:** 10.1371/journal.pone.0113501

**Published:** 2014-11-19

**Authors:** Johanna Rhodes, Mathew A. Beale, Matthew C. Fisher

**Affiliations:** 1 Department of Infectious Disease Epidemiology, Imperial College London, London, United Kingdom; 2 Institute of Infection and Immunity, St. George's University of London, London, United Kingdom; University of Minnesota, United States of America

## Abstract

The industry of next-generation sequencing is constantly evolving, with novel library preparation methods and new sequencing machines being released by the major sequencing technology companies annually. The Illumina TruSeq v2 library preparation method was the most widely used kit and the market leader; however, it has now been discontinued, and in 2013 was replaced by the TruSeq Nano and TruSeq PCR-free methods, leaving a gap in knowledge regarding which is the most appropriate library preparation method to use. Here, we used isolates from the pathogenic fungi *Cryptococcus neoformans* var. *grubii* and sequenced them using the existing TruSeq DNA v2 kit (Illumina), along with two new kits: the TruSeq Nano DNA kit (Illumina) and the NEBNext Ultra DNA kit (New England Biolabs) to provide a comparison. Compared to the original TruSeq DNA v2 kit, both newer kits gave equivalent or better sequencing data, with increased coverage. When comparing the two newer kits, we found little difference in cost and workflow, with the NEBNext Ultra both slightly cheaper and faster than the TruSeq Nano. However, the quality of data generated using the TruSeq Nano DNA kit was superior due to higher coverage at regions of low GC content, and more SNPs identified. Researchers should therefore evaluate their resources and the type of application (and hence data quality) being considered when ultimately deciding on which library prep method to use.

## Introduction

For a newcomer into the field of high-throughput genomics, the plethora of available library preparation methods, with widely contrasting sample inputs, workflows, and potential biases can be bewildering. Sequencing by synthesis, as developed by Illumina, is currently the market leader in high-throughput next-generation sequencing (NGS) methods [1], [2], [3]. Illumina has progressively improved and enhanced on its early library preparation methods, with newer methods becoming simpler and quicker to perform, whilst also yielding more consistent results. This includes the different options for shearing genomic DNA; the standard method has been ultrasonication, but since the cost of the precision ultrasonicators recommended by Illumina is substantial, this has made library preparation prohibitive to many laboratories. A more recent alternative is the use of enzymatic cleavage with or without integrated transposome insertion of adaptor sequences, as used in the Nextera and Nextera XT protocols (Illumina) [4]. However, the use of enzymes to fragment genomic DNA has been shown to contain certain GC biases leading to unequal uneven sequence coverage [5].

Further considerations include the size selection of sheared DNA: both ultrasonic and enzymatic shearing can produce libraries with sheared DNA over a range of 600 bp or more, which is unsuitable for many sequencing projects that require a very specific sequence length. Size selection allows the refinement of the sheared DNA into a very specific size range. The earlier Illumina protocols were based on gel extraction, which was time consuming and technically challenging, whilst newer methods leverage the preference of paramagnetic SPRi beads (e.g. Ampure XP; Beckman-Coulter) for binding larger DNA fragments, allowing carefully controlled sequential binding steps to remove large then small fragments from a DNA library. These methods allow the size profile of DNA libraries to be refined to within 100–200 bp [6].

More recent kits have also been designed with the limitations of the technology in mind. One of the major considerations of genome sequencing is GC induced bias [4], [7]. Theoretically, shearing by mechanical means such as ultrasonication should lead to random shearing. In contrast, cleavage by enzymatic means will be inherently biased by the location of restriction or insertion sites and by the GC content of the DNA [1]. Furthermore, protocols that incorporate PCR to enrich content, as included in most Illumina protocols, are introducing further GC-based bias as well as additional sequencing errors due to PCR amplification. This has led to the development of protocols that use polymerases less prone to GC bias and with increased amplification fidelity for PCR, or the elimination of PCR entirely (as seen in Illumina's PCR-free protocol).

The TruSeq DNA protocol has been the mainstay of genomics projects for a number of years. This method utilises a relatively high sample input concentration and, when following the manufacturer's instructions, produces useful libraries with a minimum input of 1 µg of genomic DNA (gDNA). By default, DNA is sheared by ultrasonicator and size selection is performed using gel extraction. Between 2011 and 2012, the Illumina sequencing platform was the clear market leader [8], and the TruSeq DNA method was the main method for library preparation supplied by Illumina for DNA sequencing. However, in May 2013 Illumina announced that the TruSeq DNA kits would be discontinued at the end of the year, with final shipping dates in March 2014. The withdrawal of such a well established and widely used kit may leave researchers uncertain as to which of the now wide variety of available kits that they should choose for their sequencing project. Our aim was to address this question both for ourselves and for other researchers in the field.

Here, we evaluate and compare two new library preparation methods, the TruSeq Nano DNA kit (Illumina) and the NEBNext Ultra DNA kit (New England Biolabs), against the original market leader, TruSeq DNA kit (Illumina). TruSeq Nano is marketed as having a basis in the original TruSeq DNA sample prep method, but requiring a lower input gDNA (100–200 ng). For this reason, we chose this over the TruSeq PCR-free method, which requires a similar or greater starting amount of gDNA (1–2 µg) to TruSeq DNA. NEBNext Ultra also boasts advantages such as low inputs of gDNA (5 ng), and creates indexed libraries suitable for the Illumina platform sequencing machines, and as such, is marketed as a cheaper alternative to Illumina.

The human-infecting pathogenic fungus *C. neoformans* var. *grubii* (*Cng* henceforth) is routinely sequenced in our laboratory, with DNA extraction methods optimised for whole-genome sequencing applications. As this fungus is the focus of several large-scale population genomics projects worldwide, there is a need to streamline sequencing protocols and the attendant bioinformatics pipelines in order to optimise the quality of data amongst projects. These needs are common to many laboratories aiming to sequence microbial eukaryotes with similar sized genomes to *Cng*; as such, this organism is an ideal model for reviewing library preparation methods.

## Materials and Methods

### DNA extraction

Glycerol stocks of stored *Cng* isolates were plated onto Saboroud Dextrose (SD) agar (Oxoid, Fisher Scientific) and grown at 30°C for 72 hours. Single colonies were selected and inoculated in 6 ml Yeast Peptone Digest (YPD) liquid media (Sigma-Aldrich) supplemented with 0.5 M NaCl, followed by inoculation at 37°C with agitation (165 rpm) for 40 hours. Fungal DNA was extracted using the MasterPure Yeast DNA Purification kit (Epicentre) according to the manufacturer's instructions, but with the addition of two cycles of rapid bead beating (45 seconds, 4.5 m/sec) using a RiboLyser Homogenizer (Hybaid, Middlesex, UK) and 1.0 mm silica beads (Thistle Scientific, UK) prior to the heat inactivation step. Genomic DNA was resuspended in Buffer EB (Qiagen) to avoid EDTA in the final preparation.

### Sample preparation and quality assessment

Purified DNA was quantified using the Qubit Broad Range double-stranded DNA assay (Life Technologies), and diluted in Buffer EB (Qiagen) to the concentration required for input into each library preparation protocol using a two-step, quantitation and dilution, then re-quantitation and re-dilution procedure to ensure accuracy of dilution. Selected DNA samples were assessed for quality by gel electrophoresis and Genomic DNA Screen Tapes using TapeStation 2200 (Agilent). The same genomic DNA purification was used as starting material for all three library preparation methods.

### Library preparation

Library preparations were performed according to manufacturer's instructions, in 96-well MicroAmp Optical 96-Well Reaction Plates (Life Technologies) or Hard-Shell Low-Profile Thin-Wall 96-Well Skirted PCR plates (BioRad). Quality and band size of libraries were assessed using D1K and HS D1K Screen Tapes (Agilent) on a Tapestation 2200 (Agilent) at multiple steps during each protocol, typically after size selection and after PCR amplification. Libraries were quantified by qPCR using the Library Quantification Kit for Illumina sequencing platforms (KAPA Biosystems, Boston, USA), using a Prism 7300 Real Time PCR System (Life Technologies). Unless otherwise stated, libraries were normalised to a working concentration of 10 nM using the molarity calculated from qPCR adjusted for fragment size with the Tape Station median.

### TruSeq DNA v2

Input genomic DNA (gDNA) was used at concentrations between 50 ng/µl and 150 ng/µl for the TruSeq DNA v2 protocol. Fifty-four microlitres of gDNA was transferred to an AFA fiber Snap-Cap microTUBE (Covaris) and sheared on an S2 Ultrasonicator (Covaris) with a Duty Cycle of 10%, Intensity set to 5.0, 200 cycles per burst, in frequency sweeping mode for 50 seconds. Library preparation was performed according to the manufacturer's instructions, with size selection performed using Tris-Borate-EDTA (TBE) agarose gel electrophoresis and MinElute Gel Extraction (Qiagen). Adaptor enrichment was performed using ten cycles of PCR according to the manufacturer's instructions.

### TruSeq Nano DNA

Genomic DNA for input into the TruSeq Nano DNA protocol was quantified and diluted to 2 ng/µl. Fifty-four microlitres of gDNA was sheared using an S2 Ultrasonicator (Covaris) using the same settings as for the TruSeq DNA protocol. Library preparation was performed according to the manufacturer's instructions. Adaptor enrichment was performed using eight cycles of PCR according to the manufacturer's instructions.

### NEBNext Ultra DNA

Genomic DNA for input into the NEBNext Ultra DNA protocol was quantified and diluted to 2 ng/µl. Fifty-four microlitres of gDNA was sheared using an S2 Ultrasonicator (Covaris) using the same settings as for the TruSeq DNA protocol. Library preparation was performed according to the manufacturer's instructions, with size selection performed using AMPure XP beads (45 µl beads for the initial step, and 25 µl for the second step). Adaptor enrichment was performed using eight cycles of PCR, and using the NEBNext Multiplex oligos for Illumina (New England Biolabs).

### Sequencing

TruSeq DNA v2 libraries were pooled in groups of ten per lane, whilst TruSeq Nano and NEBNext Ultra libraries were pooled in groups of eight per lane, and run with paired-end 100 bp reads on a HiSeq 2000 set to high yield mode at MRC Clinical Genomics Centre (Hammersmith, London, UK). Libraries prepared by the same method were sequenced on the same lane of a flow-cell, but the different methods were sequenced on different flow cells. All raw reads and information on lineages of isolates in this study have been submitted to the European Nucleotide Archive, under the project accession PRJEB7411.

### Read alignment

All reads were mapped to the reference genome using an identical pipeline. Reads were mapped to the *Cng* reference genome, H99 [7] using BWA 0.75a [10] aln and quality threshold of 15. Samtools [11] version 0.1.18 was used to sort and index resulting BAM files, and generate information about the alignment output. Picard [12] version 1.72 was used to locate duplicate reads and assign correct read groups to BAM files. All resulting BAM files were recalibrated by locally realigning around INDELs using GATK RealignerTargetCreator and IndelRealigner [13].

### SNP and INDEL detection

SNPs and INDELs were called from all alignments in the same way, using GATK UnifiedGenotyper [14], [15] version 2.2-2 in haploid mode with a downsampling value of 10000. Both SNPs and INDELs were filtered according to mapping quality and read depth at each base. Any SNPs or INDELs not present in at least 80% of reads were also filtered out. SNPs were also called using bcftools [16] to confirm SNP numbers called using GATK.

### Genome coverage

BAM files locally realigned around INDELs were used to determine the average (mean) coverage, using GATK [13] DepthOfCoverage package and default settings. The *Cng* H99 genome [9] was again used as the reference. IQR values were calculated using the MATLAB ‘iqr()’ function (release 2011b, The MathWorks Inc., Natick, MA). Coverage gaps were identified and counted using a custom MATLAB script.

### GC-content analysis

The ‘CollectGcBiasMetrics.jar’ package, part of the Picard [12] software was used to collect information about GC bias in the reads of BAM file by counting the number of reads in each 100 bp window using default settings, and therefore providing a measure of coverage relating to GC content.

## Results

The pathogenic fungus *Cng*, an organism with a genome of approximately 19 Mb in length, and a GC content of 48.23%, is routinely whole-genome sequenced and aligned to the reference strain in our laboratory. We randomly selected an isolate (VNI molecular type), which has been sequenced using various library prep methods, and sequenced this isolate using the TruSeq DNA v2 kit (Illumina), and two newer kits: the TruSeq Nano DNA kit (Illumina), and the NEBNext Ultra DNA kit (New England Biolabs). We expanded the range of isolates tested using the newer kits by randomly selecting a further four isolates (three of the VNI molecular type, one of VNII), which represent the span of known SNP diversity present in *Cng* genomes.

All samples were sequenced by HiSeq 2000, and the resulting reads and genome assemblies were compared. All resulting reads generated were mapped to the reference *Cng* genome, H99 [9] as described in Methods. Our aim was to determine firstly if the newer library preparation kits were equivalent or better than the existing TruSeq DNA v2 kit, and secondly which of the two newer kits performs better in terms of library quality and depth, but also cost, ease of use, and time.

### Cost

Any comparison of cost is subject to both local variation and the constantly changing prices of competitive pricing strategies, and as such this information may be out dated very rapidly. In particular, at the time of writing, the Illumina TruSeq DNA v2 has been discontinued, so performing direct price comparisons is difficult. Never-the-less, certain comparisons may be made between the two current methods as the differences may not be obvious to the newcomer.

Whilst both kits contain most of the reagents required to perform library preparation, the difference between the kits is in the additional components that need to be purchased. For both methods, it is advisable to perform quantitation and dilution of genomic DNA prior to beginning, and both methods require shearing by ultrasonication, necessitating the separate purchase of Covaris tubes (∼£4.80 GBP/sample), and access to a Covaris Ultrasonicator. From this point on, the TruSeq Nano kit provides a near complete solution, including nearly all the reagents required to perform a complete library preparation up to final quality control and normalisation. In contrast, the NEBNext Ultra kit does not include all reagents, instead allowing users the flexibility to select the methods and reagents most appropriate to their investigation. This means that the quoted cost of the NEBNext Ultra kit is incomplete – additional purchases such as oligonucleotide primers with Illumina Index sequences for multiplexing and SPRi beads for library purification and size selection (Ampure XP beads), are required. Further considerations include the increased number of bead-based purification steps included in the TruSeq Nano kit (five, versus two in the NEBNext Ultra protocol) – during large library preps these extra steps significantly increase the usage of sterile, filtered pipette tips. Whilst following both protocols carefully, we estimated these additional clean-up steps (and other steps) accounted for an additional 29 tips per sample (or 660 per 24 sample kit). Overall, the cost of library prep is very similar between the two kits (Table 1); by our estimates, the NEBNext Ultra kit is marginally the cheaper of the two by approximately £1 per sample (or £30 per 24 samples).

**Table 1:** Cost comparison for library prep consumables, based on UK list prices (May 2014) where possible.

	TruSeq Nano		NEBNext Ultra	
Consumables	Per 24	Per sample	Per 24	Per sample
Core library prep kit	£812	£31.47	£640	£26.67
Additional oligos	£0	£0	£121	£5.04
Ampure XP beads	£0	£0	£44	£1.83
Covaris tubes	£110	£4.58	£110	£4.58
Quality control analysis	£200	£8.33	£200	£8.33
Filter tips (assuming £35/1000)	£71	£3.12	£48	£2.10
Total	£1193.40	£49.87	£1163.30	£48.56

Quality control analysis included quantification of all samples by Qubit Broad Range dsDNA assay (Life Technologies) prior to beginning, follow by final analysis using TapeStation 2200 D1K Screen Tapes (Agilent) and qPCR using the Kapa kit for Illumina libraries (Kapa Biosciences). Filter tip and Ampure XP bead costs are based on estimates of usage, with ‘per sample’ usage rounded up to the nearest tip.

### Time and ease of use

Although Illumina publicise estimated time to complete a library preparation, these times are typically given for a very small numbers of samples. In our laboratory, we routinely prepare libraries in batches of 24 samples, and find it takes considerably longer. The original TruSeq DNA v2 protocol required gel extraction, including running samples on agarose gels for up to two hours – with 24 samples it may be necessary to run as many as four such gels. This labour intensive process could extend library preparation by a day or more. The replacement of gel extraction of libraries for size selection with SPRi bead selection in the two newer methods is a great time-saving improvement, and significantly streamlines workflow.

In our hands, 24-sample library prep takes approximately 2 days to complete using the newer protocols. Both TruSeq Nano and NEBNext Ultra methods have very similar work flows, and rely on SPRi bead-based size selection. Incubation times are similar for most steps. The Illumina protocol adds index sequences during adaptor ligation, whilst the NEBNext Ultra protocol adds indexes to adaptor tagged fragments during the PCR enrichment steps, but these differences do not significantly impact on workflow. The primary workflow difference between the two methods is the reduced number of SPRi purification steps with the NEBNext kit (including size selection, the TruSeq Nano protocol requires five bead purifications, whilst the NEBNext Ultra requires only two). Each of these steps takes approximately 30 mins for a 24-sample protocol, resulting in a time saving of at least 90 minutes for the NEBNext kit. Therefore, of the two kits NEBNext Ultra is faster, but only marginally so.

### Data quality from the two newer methods is greater than that generated by the older TruSeq DNA v2 method

We first investigated the reads obtained from an isolate that was sequenced using all three library preparation methods. We assembled the reads and looked at read depth, SNP and INDEL calling, and genome coverage and GC bias.

#### Both TruSeq Nano and NEBNext Ultra yield more SNPs compared to TruSeq DNA v2

In population-based studies, the calling of SNPs and insertions and deletions (INDELs) are important for the discovery of genetic variation between individuals within a population. Over- or underestimating diversity can also influence the results of downstream analyses, such as recombination detection and population genetic structure. Therefore, there is a strong need for variant calling to be accurate. Errors in variant calling can lead to false positive SNPs being identified, or true positives being unaccounted for. High false positive rates would lead to extra validation being required, such as additional sequencing, which increases the amount of time and money spent to identify variants.

**Figure 1:**
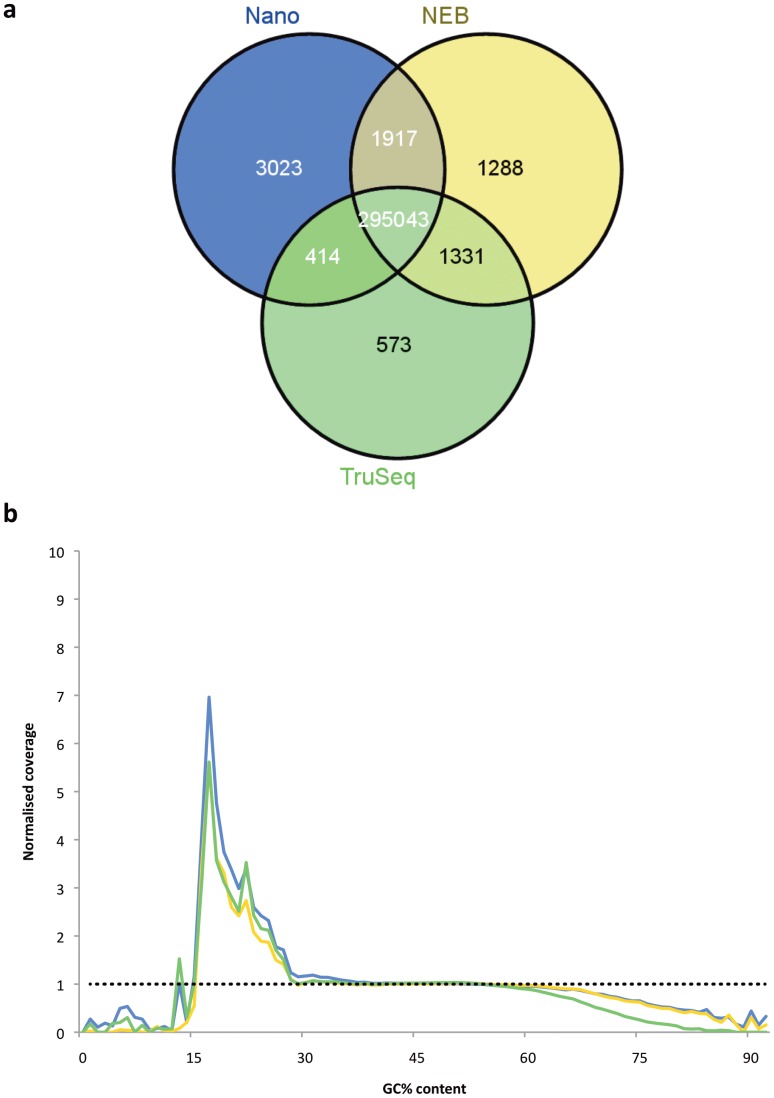
New library prep methods perform better than TruSeq DNA. a) Varying numbers of SNPs are found to be in common between the two newer library prep methods, and the original TruSeq DNA v2 kit. The majority of called SNPs were common to both of the newer library prep methods, and the original TruSeq DNA v2 kit; whilst both TruSeq Nano (blue) and NEBNext Ultra (yellow) performed better than the original TruSeq DNA v2 (green), with a larger number of SNPs called against a reference, a greater number of SNPs were uniquely called in the TruSeq Nano dataset along (blue). Venn diagrams were generated using the Venny software [17] of SNPs called using GATK [14], [15]. b) Both NEBNext Ultra and TruSeq Nano exhibit higher coverage in GC-rich regions compared to the original TruSeq DNA v2 kit. Normalised coverage (binned into 100 bp windows) relating to GC content, where the blue line represents the TruSeq Nano-prepared isolates, the green line represents the TruSeq DNA v2-prepared isolate, and the yellow line represents the NEBNext Ultra-prepared isolate. The black dotted line at *x* = 1 is the expected normalised coverage showing no bias. Whilst all library preparation methods perform similarly, at GC-rich regions the newer library prep methods yield higher coverage than the original TruSeq DNA v2 method.

Variants were called against the *Cng* H99 reference genome [9] using the Genome Analysis Toolkit (GATK; The Broad Institute) UnifiedGenotyper, and filtered based on mapping quality and read depth, as described in Methods. Firstly, we investigated the reads from the isolates sequenced with the TruSeq DNA v2, TruSeq Nano (both Illumina) and NEBNext Ultra DNA kit (New England Biolabs). More true positive SNPs were called in isolates prepared with the two newer methods, compared to the original TruSeq DNA v2 method (Figure 1a). Comparison of the false positive rates for each library prep method (Table 2) indicate that SNPs are more likely to be incorrectly identified in the newer methods; however, the number of true positive SNPs identified (i.e. those that have fulfilled the filtering criteria) is ultimately higher in the newer methods, compared to the original TruSeq DNA method.

**Table 2:** SNP calls from two different pipelines and false positive rates associated with calling SNPs against the *Cng* reference [9].

Call platform	Call dataset	Called SNPs (GATK [13], [14])	Called SNPs (bcftools [15])	Filtered SNPs (GATK [13], [14])	False positive rate (%)
TruSeq DNA	CN-3	302435	283221	297361	1.68
	CN-1	52341	50033	49215	5.97
	CN-2	33378	31533	31483	5.67
TruSeq Nano	CN-3	306623	289467	300397	2.03
	CN-4	50556	48115	47599	5.85
	CN-5	11664	10938	10844	7.03
	CN-1	51837	49864	48804	5.85
	CN-2	33141	31322	31350	5.40
NEBNext Ultra	CN-3	305659	287502	299579	1.99
	CN-4	50134	47838	47425	5.40
	CN-5	11415	10042	10738	5.93

The converse, however, is true for INDELs: whilst the false positive rate remained the same for all three library prep methods, more true positive INDELs were called in the same isolate prepared with TruSeq DNA, compared to those prepared with NEBNext Ultra and TruSeq Nano (Table 3).

**Table 3:** False positive rates associated with called INDELs against the *Cng* reference [9].

Call platform	Call dataset	Called INDELs	Filtered INDELs	False positive rate (%)
TruSeq DNA	CN-3	28690	28676	0.05
	CN-1	5368	5354	0.26
	CN-2	3490	3476	0.40
TruSeq Nano	CN-3	26509	26495	0.05
	CN-4	5293	5279	0.26
	CN-5	1407	1393	1.00
	CN-1	5278	5264	0.27
	CN-2	3468	3454	0.40
NEBNext Ultra	CN-3	26233	26219	0.05
	CN-4	5225	5211	0.27
	CN-5	1391	1377	1.01

#### Genome coverage and GC bias

Depth of coverage, as described in Methods, was found to be lower in the isolate prepared with TruSeq DNA v2, compared to the same isolate prepared with the two newer methods (Table 4). When analysing genome coverage statistics, low inter-quartile ranges (IQRs) are indicative of uniform coverage across the genome; a high IQR is indicative of non-uniform coverage. The isolate prepared using the TruSeq DNA kit was found to have a significantly higher IQR than the same isolate prepared with the newer kits (Table 4). Indeed, the TruSeq DNA v2-sequenced isolate was also found to have a greater number of bases at low and zero coverage (Table 4). Together, this indicates that the two newer library prep methods have not only improved the amount of coverage, but also the uniformity of coverage, and therefore, perform better than the original TruSeq DNA v2 kit.

**Table 4:** IQR of read depths of TruSeq Nano and NEBNext Ultra prepared samples.

Call platform	Call dataset	Mean coverage	IQR	Bases at low coverage (<15×)	Bases at zero coverage
TruSeq DNA	CN-3	80	22	4.81%	2.12%
	CN-1	152	5	1.41%	0.79%
	CN-2	191	4	1.26%	0.83%
TruSeq Nano	CN-3	148	4	3.89%	1.46%
	CN-4	163	4	1.07%	0.59%
	CN-5	192	3	0.42%	0.22%
	CN-1	112	9	1.63%	0.93%
	CN-2	193	7	1.29%	0.86%
NEBNext Ultra	CN-3	158	6	4.06%	2.87%
	CN-4	159	4	1.15%	0.62%
	CN-5	146	7	0.49%	0.26%

This finding was supported by the GC bias on genome coverage exhibited by the TruSeq DNA v2 isolate: at GC-rich regions, the TruSeq DNA v2 isolate was seen to have less coverage, compared to the newer library prep methods (Figure 1b).

### Both NEBNext Ultra and TruSeq Nano methods have advantages suitable for a replacement to TruSeq DNA v2

After investigating the efficacy of the new methods over the discontinued ‘gold standard’ method TruSeq DNA v2 method, we then proceeded to a more in depth comparison between the two new methods.

#### Both TruSeq Nano and NEBNext Ultra yield more SNPs compared to TruSeq DNA v2

**Figure 2:**
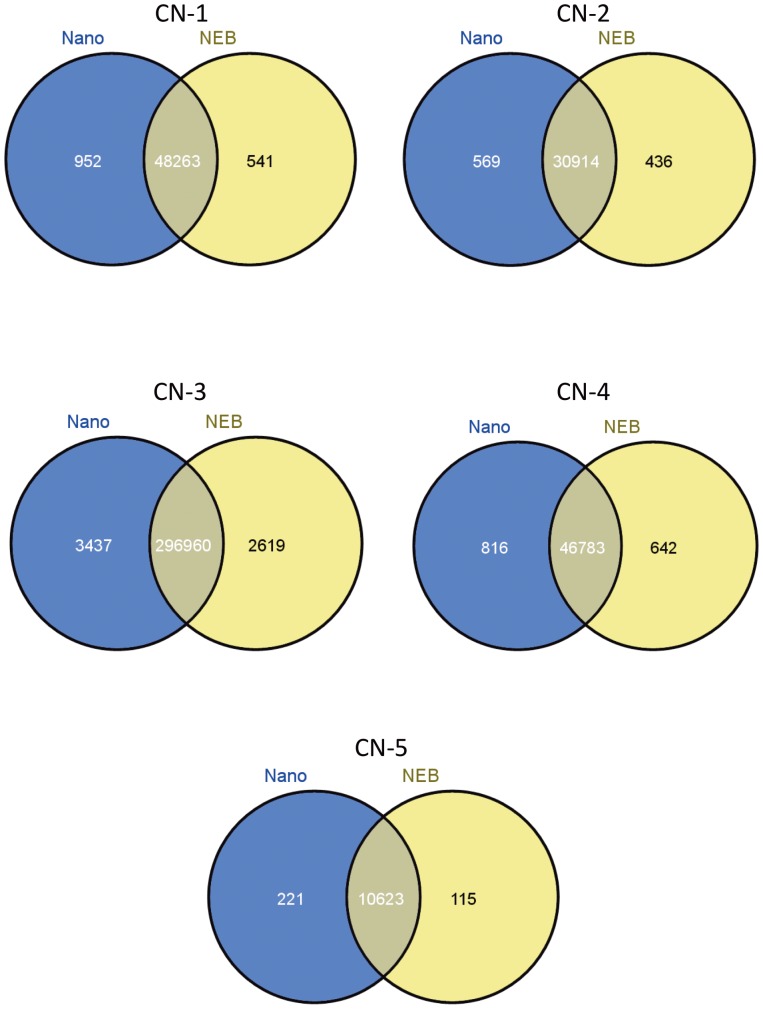
Uniquely and commonly called SNPs in TruSeq Nano and NEBNext Ultra-prepared isolates. The majority of called SNPs were common to both methods in each isolate. However, a greater number of SNPs were unique to the isolate prepared with the TruSeq Nano method (blue). Venn diagrams were generated using the Venny software [17] of SNPs called using GATK [14], [15].

The false positive rate for SNP calling is higher in genomes prepared with TruSeq Nano compared to NEBNext Ultra (Table 2); however, this is not the case when calling INDELs (Table 3). Despite this, more filtered, high confidence SNPs were identified in the isolates prepared with the TruSeq Nano DNA kit, compared to those prepared with the NEBNext Ultra kit. Further investigation revealed that more unique SNPs were called in the isolates prepared with the TruSeq Nano kit (Figure 2) suggesting that not all SNPs are accurately called in isolates prepared with NEBNext Ultra.

#### Genome coverage and GC bias

To compare the uniformity of coverage across the genome in both TruSeq Nano and NEBNext Ultra library-prepared isolates, we measured the depth of coverage, as described in Methods.

**Figure 3:**
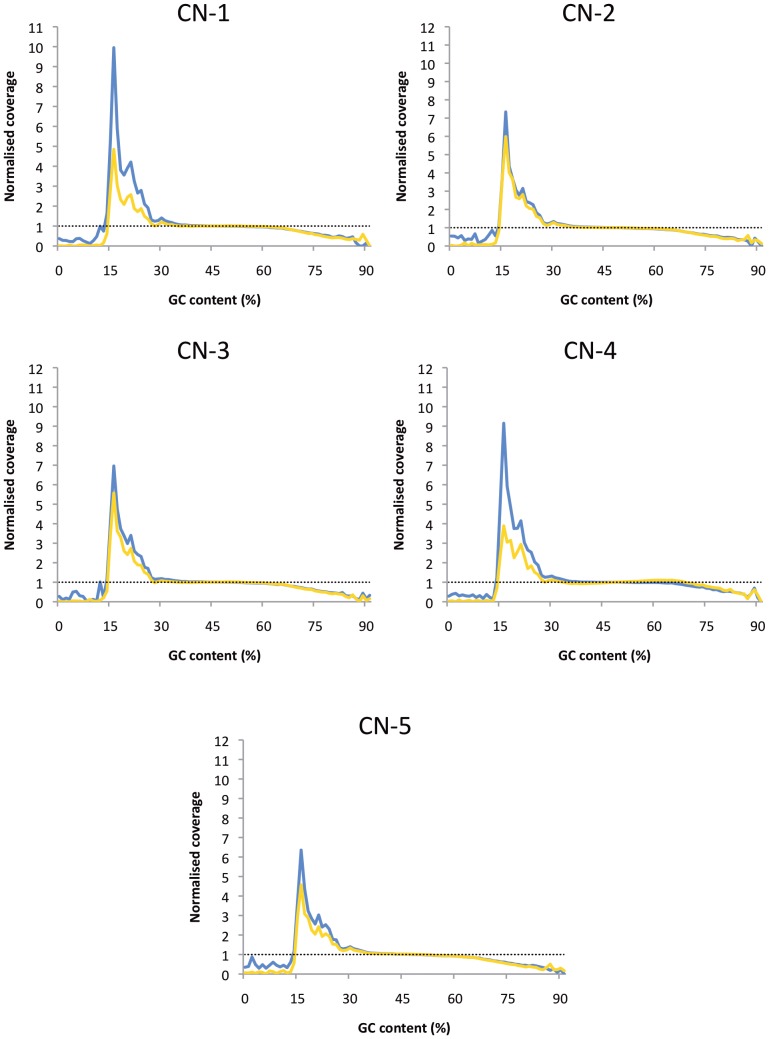
Coverage is biased at AT- and high GC-rich regions. Normalised coverage (binned into 100 bp windows) relating to GC content, where the blue line represents the TruSeq Nano-prepared isolate, and the yellow line represents the NEBNext Ultra-prepared isolate. The black dotted line at *x* = 1 is the expected normalised coverage showing no bias. Whilst both library preparation methods perform similarly, the TruSeq Nano-prepared isolates generally provide more coverage at AT-rich regions.

In our data, both library preparation methods were capable of providing deep coverage (Table 4). However, higher inter-quartile ranges (IQRs) were observed in the coverage of NEBNext Ultra genomes, compared to the same genome prepared using TruSeq Nano (Table 4). A high IQR was observed for the NEBNext Ultra isolates, suggesting that this library prep method does not provide a uniform coverage; a more uniform coverage is seen with the TruSeq Nano-prepared genomes. This was also evident when genome coverage was plotted against percentage GC content (Figure 3): coverage dropped more severely at high AT regions for isolates prepared with the NEBNext Ultra kit, however, both kits performed equally poorly at regions with high GC content.

Gaps in coverage, defined as any bases or regions of the genomes that are sequenced with less than 15% read depth, provide a meaningful way to look at non-uniform sequence coverage. Isolates prepared with the TruSeq Nano DNA kit again display statistically significant (*p*<0.016) more uniform coverage, with fewer gaps seen along the genome, compared to the isolates prepared with the NEBNext Ultra DNA kit (Table 4).

## Discussion

With the discontinuation of the market leader for library preparation methods, Illumina's TruSeq DNA v2 kit, a gap has been created for a new method to become the most widely used kit. A fundamental feature of all library preparation methods for NGS is their speed, with decreasing laboratory and machine run time. The workflow for NEBNext proved to be quicker by approximately 90 minutes, but in the context of a two-day protocol, this is not a large difference. The cost comparison showed that for a 24-sample preparation, at current prices the NEBNext Ultra kit is also less expensive by £30.10, but again, in the context of an £1100 protocol, this is insubstantial. Therefore we cannot recommend one kit over another based solely on workflow and cost.

The ability to call SNPs is very similar for both the Illumina and NEB methods; however, detection of SNPs in isolates prepared using the NEBNext Ultra kits was not as accurate. This finding was confirmed when using a different SNP caller (bcftools [16]), suggesting there is an underlying difference in the data generated, not the bioinformatics pipeline used (Table 2). One would also desire high depth of coverage for stringent SNP detection: whilst one library preparation method did not outperform the other, the uniformity of coverage was more preferable in those isolates prepared using the Illumina TruSeq Nano DNA kit.

Sequencing of microbial genomes is subject to many caveats. Culturing the same colony for extraction on separate occasions may result in the generation of random mutations which lead to slightly altered consensus sequences. Library preparation may be subject to biases such as pipetting accuracy, extended incubation times, and PCR induced SNPs. Furthermore, variations in flow-cell clustering on HiSeq may lead to biases both between flow-cells and between lanes. For this study, we used the same genomic DNA purifications for each library preparation to minimize culture bias. The library preps and sequencing were only performed singly, but were performed by the same experienced person. Ideally, one would wish to repeat these library preparations and repeat the sequencing across multiple lanes of HiSeq in order to control for both library prep variability and lane bias. However, this was not possible due to cost and time constraints. Therefore we appreciate that some of the differences between methods may be the result of library prep and lane biases. However, in our laboratory we routinely include sample CN-5 with all 24-sample library preps, and therefore have a large number of replicates available using the TruSeq Nano protocol across many lanes and flow-cells of HiSeq. We have found the combined effects of library prep and lane bias to be low with this sample, with the Venn of 6 replicates containing 10,496 common SNPs and 142 unique SNPs (data not shown). Furthermore, we only performed sequencing of five isolates from a single organism of moderate GC content, and although interesting observations may be made, a larger sample size would be necessary to allow comprehensive comparisons between methods.

Coverage can be misleading and is more likely to be ambiguous for reads spanning repetitive regions of the genome, which includes regions of high AT and GC content. Ultimately, this can cause problems when aligning reads to a reference genome or for *de novo* assembly. Whilst steps are made to optimise the PCR amplification of the library, bias in coverage was still seen at regions with high GC content (Figure 3), with neither method preferable.

The uniformity of coverage, and reduced GC content bias seen in isolates prepared with the Illumina TruSeq Nano DNA kit suggest that in terms of data accuracy, this would be the ideal replacement for the resequencing of small microbial eukaryote genomes, and a potential market leader, to the now discontinued TruSeq DNA kit.
